# 3-(4-Hydroxyphenyl)propionic acid, a major microbial metabolite of procyanidin A2, shows similar suppression of macrophage foam cell formation as its parent molecule

**DOI:** 10.1039/c7ra13729j

**Published:** 2018-02-07

**Authors:** Yu-Ying Zhang, Xiao-Le Li, Tong-Yun Li, Mei-Ying Li, Ri-Ming Huang, Wu Li, Rui-Li Yang

**Affiliations:** Guangdong Provincial Key Laboratory of Food Quality and Safety, College of Food Science, South China Agricultural University Guangzhou 510642 China rlyang77@scau.edu.cn +86-20-85280270 +86-20-85283448; College of Food Science and Technology, Hainan University Haikou 570228 China leewuu@163.com +86-898-66193581 +86-898-66198861

## Abstract

The effect of procyanidin A2 (PCA2) and its major colonic metabolite 3-(4-hydroxyphenyl)propionic acid (HPPA) on the suppression of macrophage foam cell formation, and underlying mechanism, were investigated for the first time. The results showed that 12.5 μg mL^−1^ PCA2 and HPPA significantly reduced cellular lipid accumulation and inhibited foam cell formation. HPPA promoted macrophage cholesterol efflux by up-regulating mRNA expressions of ABCA1 and SR-B1, while PCA2 significantly increased SR-B1 and LXR-α mRNA expression levels. Moreover, PCA2 and HPPA significantly lowered the elevated levels of CD36 mRNA expression in ox-LDL-treated macrophage cells. Besides these, the ox-LDL-induced cellular oxidative stress and inflammation was also restricted by PCA2 and HPPA treatment *via* nuclear factor kappa-B pathways. In conclusion, PCA2 and its major microbial metabolite, HPPA, inhibited the conversion of macrophage into foam cells *via* regulating cellular lipid metabolism and suppressing cellular oxidative stress and inflammation.

## Abstract 2

PCA2 and its major microbial metabolite HPPA inhibited macrophage foam cell formation, which may be due to regulating ABCA1, SR-B1 and CD36 expression, and restricted cellular oxidative stress and inflammation via NF-κB pathway.

## Graphical abstract

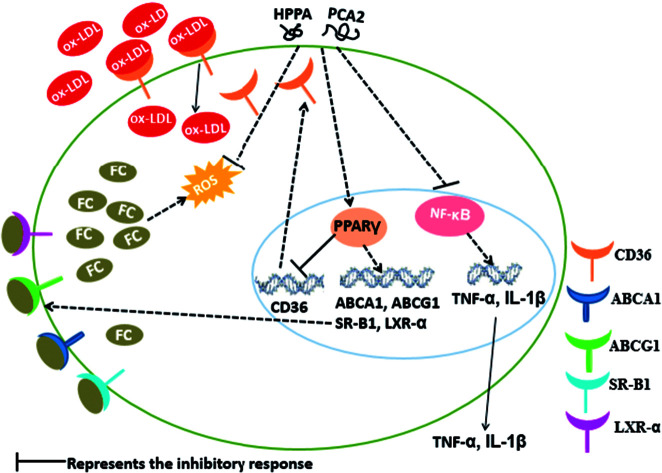

## 1. Introduction

Atherosclerosis (AS), a leading cause of death and illness in the world, has been considered to be an important pathological basis of cardiovascular disease. Excessive uptake of oxidized low-density lipoprotein (ox-LDL), excessive cholesterol esterification, and impaired cholesterol efflux occurring in the macrophages, results in the formation of foam cells.^1^ It has been shown that the conversion of macrophages into foam cells is a typical pathological feature of early stage atherosclerotic lesions.^2^ Therefore, suppressing macrophage derived foam cell formation is critical as an effective strategy for prevention and treatment of AS.

In order to develop novel therapeutic interventions for AS, the mechanisms underlying the formation of foam cells have been studied in recent years.

Reverse cholesterol transport (RCT) is the only way to remove excess cholesterol, which is important for the removal of excess lipids from peripheral tissues to the liver for catabolism and excretion.^3^ The most important step in macrophage RCT is cholesterol efflux, by which intracellular cholesterol from macrophages is transferred to extracellular acceptors. ATP-binding cassette transporter A1 (ABCA1), ATP-binding cassette transporter G1 (ABCG1) and scavenger receptor BI (SR-BI) play key roles in RCT pathway.^4^ Studies have shown that the expressions of ABCA1 and ABCG1 are to some extent, regulated by peroxisome proliferator-activated receptor gamma (PPARγ) activators and liver X receptor alpha (LXR-α).^5^ CD36, a high affinity physiological receptor for ox-LDL, plays a vital role in the foam cell formation. ox-LDL can stimulate CD36 expression, then the up-regulation of CD36 make more ox-LDL endocytosed, and eventually lead to excess cholesterol buildup to form foam cells.^6^ Growing evidence establishes that the imbalanced expressions of CD36, ABCA1, ABCG1 and SR-B1 mediate the foam cell formation *via* impairing the balance of cholesterol influx and efflux.^7^ Furthermore, oxidative stress and inflammation play important roles in the pathogenesis of diverse cardiovascular diseases including AS. Macrophages can phagocytosed ox-LDL and other lipids and produce inflammatory cytokines and reactive oxygen species (ROS) while continuing to accumulate lipids and differentiate into foam cells to form the early atherosclerotic plaques.^8^

Procyanidins, which are among the most abundant dietary polyphenols, are recognized as biologically active in human health. Procyanidins have strong anti-oxidant,^9^ anti-inflammation,^10,11^ anti-aging capabilities^12^ and can help prevent cardiovascular and cerebrovascular diseases. The subunits of procyanidins are linked by C4–C8 or C4–C6 bond (B-type), or doubly linked by a C4–C8/C6 bond and an additional C2–O–C7 or C2–O–C5 ether bond (A-type). Moreover, the most ubiquitous procyanidins in foods are the B-type procyanidins, the A-types are less common and concerned in nature.^13^ B-type procyanidins mainly from apple,^14^ grape seed,^15^ cocoa^16^ exhibited great health benefits for preventing the development of AS. Recently, it is indicated that A-type procyanidins similarly have some different bioactivities, such as effective uropathogenic bacterial anti-adhesion activity.^17,18^ Wong *et al.*, reported that the deleterious effect of *p*-cresol on HT29 cells were prevented by the extracts from cranberries and avocados (characterized by the presence of type-A procyanidins), whereas they became worse by extracts from apples and grape.^19^ However, the effect of A-type procyanidins on foam cells formation remains unclear.

Though potential health beneficial effects have been attributed to procyanidins, it is poorly absorbable in the gastrointestinal tract. Current evidences indicate that colonic microbiota can degrade procyanidins, producing a large number of metabolites that can be absorbed and exert biological effects in the body.^20^ In particular, colonic metabolites have demonstrated anti-oxidant,^21^ anti-inflammatory,^22^ and anti-glycative^23^ activities. Altogether, these findings support the hypothesis that not only procyanidins but also their microbial metabolites must be taken into account when assessing the impact of procyanidins on health.^24^ However, up to date, whether the microbial metabolite of procyanidin has an inhibitory effect on foam cells formation is unclear.

As mentioned above, most of studies assessing the anti-atherosclerosis activity of procyanidins have been performed using plant procyanidins extracts (mainly composed of B-type procyanidins). We sought to determine whether A-type procyanidins and its major microbial metabolite have a direct effect on the formation of foam cells. Procyanidin A2 (PCA2) is one structurally defined A-type procyanidin that is predominately found in cranberries,^25^ avocado,^26^ peanut red skins^27^ and litchi fruit pericarp,^28^ which has been found to anti-oxidant and anti-inflammatory activities.^29^ In this study, for the first time, we investigated the interference effects of PCA2 in comparison with 3-(4-hydroxyphenyl)propionic acid (HPPA), its major microbial metabolite^30^ on foam cells formation induced by ox-LDL. The aim of this study was to investigate the potential atheroprotective effects of A-type procyanidins and its major microbial metabolite, and to gain insights into the underlying molecular mechanisms.

## 2. Materials and methods

### 2.1. Chemicals and reagents

Procyanidin A2 and 3-(4-hydroxyphenyl)propionic acid were purchased from Sigma-Aldrich (Shanghai, China). RPMI-1640 medium, DMEM medium and fetal bovine serum were purchased from Gibco Life Technologies (California, USA). Human ox-LDL was purchased from Yiyuan Biotechnologies (Guangzhou, China). The kits for total cholesterol (TC) and free cholesterol (FC) measurement were purchased from Applygen Technologies Inc., (Beijing, China). A ROS kit was purchased from Beyotime Institute of Biotechnology (Shanghai, China). The kit for determination of MDA equivalent level was purchased from Nanjing Jiancheng Bioengineering Institute (Nanjing, China). The ELISA kits of IL-6 and IL-1β were obtained from R&D System (Minneapolis, MN, USA). SYBR-Green qPCR Master Mix and RevertAid First Strand cDNA Synthesis Kit were obtained by TransGen Biotech (Beijing, China). The primary antibodies against NF-κB-p65 and β-actin were obtained from Cell Signal Technology Inc. (Beverly, MA). All other reagents were of analytical grade and obtained locally.

### 2.2. Cell line and cell culture

The mouse RAW264.7 cells were donated by Zhongshan School of Medicine, Sun Yat-sen University, Guangzhou, China. The cells were cultured in DMEM medium containing 10% fetal bovine serum (FBS) at 37 °C under 5% CO_2_.

### 2.3. Cytotoxicity assay of PCA2 and HPPA

The effect of PCA2 and HPPA on the viability of RAW264.7 cells was measured using a cell counting kit-8 (CCK-8) assay (SAB, USA) according to the manufacturer's instructions. The cells were seeded at a density of 5 × 10^4^ cells per mL and incubated with PCA2 and HPPA at the concentrations of 6.25, 12.5, 25, 50, and 100 μg mL^−1^ for 24 h. After incubation, 10 μL of CCK-8 solution was added to each well. The cells were incubated for 1 h at 37 °C and the absorbance at 450 nm was measured.

### 2.4. Oil red O staining and cellular cholesterol analysis

The raw264.7 macrophages were collected at the logarithmic growth phase and inoculated into the 6-well plates at 5 × 10^6^ cells per mL. After cells were treated with 80 μg mL^−1^ ox-LDL in the presence or absence of PCA2 and HPPA for 48 h, the cells were collected, washed three times with PBS buffer and subjected to oil-red O staining^31^ or harvested to determine the cellular cholesterol content according to the kit's instructions. The stained cells were observed and photographed under a microscope (Olympus, Tokyo, Japan). The TC and FC in cells were measured using the commercial kits according to the manufacturer's instructions. The difference between TC and FC represents the production of cellular cholesteryl ester (CE).

### 2.5. ROS and thiobarbituric acid reactive substances (MDA) assay

After washing with PBS, cells were suspended in 10 μM 2′,7′-dichlorofluorescein diacetate (DCFH-DA) solution (Beyotime, Shanghai, China) and incubated at 37 °C for 30 min. Intracellular ROS generation was monitored by measuring the fluorescence intensity of cells with an EnSpire® Multimode Plate Reader, with excitation and emission wavelengths set at 488 and 525 nm, respectively. The level of thiobarbituric acid reactive substances (TBARS, reported as MDA equivalents) was measured using a kit according to the manufacturer's instructions.

### 2.6. Determination of IL-6 and IL-1β

The cells were plated into a 96-well plate at 5 × 10^4^ cells per well and incubated with 80 μg mL^−1^ ox-LDL in the presence or absence of PCA2 and HPPA for 24 h. The cell liquid supernatant was collected by centrifugation at 5000*g* for 10 min. The levels of IL-6 and IL-1β were measured using ELISA kit (R&D System, Minneapolis, MN, USA).

### 2.7. RT-QPCR analysis

The mRNA levels of the cholesterol-metabolism-related genes were determined by real-time quantitative PCR. Total RNA was extracted and transcribed into cDNA using a Reverse Transcription System kit (TransGen Biotech, Beijing, China). The PCR primers sequences used are listed in Table 1. Real-time PCR were performed with the incorporation of SYBR green using the real-time PCR System 7500 (Applied Biosystems, Waltham, MA). Melting curve analysis and agarose gel electrophoresis were used to monitor synthesis of the PCR products. β-Actin was used as an internal control.

**Table 1:** Primer sequences of target genes and internal reference (β-actin)

Genes	Sequence (5′–3′)	Product size (bp)
β-Actin	F: GTCCCTCACCCTCCCAAAAG	202
	R: GCTGCCTCAACACCTCAACCC	
ABCA1	F: CCCAGAGCAAAAAGCGACTC	136
	R: GGTCATCATCACTTTGGTCCTTG	
ABCG1	F: AATGTCTGCTTTGCCTCGTT	108
	R: GCAGCTACTGCATGTGATCAAGA	
SR-B1	F: TTTGGAGTGGTAGTAAAAAGGGC	71
	R: TGACATCAGGGACTCAGAGTAG	
LXR-α	F: CAGCGTCCATTCAGAGCAAGTGT	140
	R: GTCAGTGAGCCTTCGCCATGTG	
PPARγ	F: GCAGCTACTGCATGTGATCAAGA	214
	R: GTCAGCGGGTGGGACTTTC	
CD36	F: GTGCTCTCCCTTGATTCTGC	102
	R: CTCCAAACACAGCCAGGAC	

### 2.8. Western-blot analysis

RAW264.7 cells were plated in 6-well plates at a density of 1 × 10^6^ cells per well. After PCA2 and HPPA treatment, the cells were washed twice with ice-cold PBS, and then were lysed with RIPA lysis containing a protease inhibitor. Protein quantification was determined using the BCA assay. Then, protein samples were electrophoresed on 8% SDS-PAGE and transferred to a PVDF membrane. Each membrane was blocked for 2 h with 5% (w/v) skimmed milk, followed by incubation with primary antibodies overnight at 4 °C. After washing five times with TBST for 5 min, the membrane was incubated for 1 h with the secondary antibody, and then washed too. The immuno-reactive protein bands were quantified by UVP ChemiDoc-IT 510 imaging system (USA).

### 2.9. Statistical analysis

Data are expressed as mean ± SD standard deviation. Significance of differences between groups was calculated by one-way ANOVA. *P* values <0.05 was considered statistically significant.

## 3. Results

### 3.1. PCA2 and the microbial metabolite HPPA promoted the proliferation of macrophages

**Fig. 1:**
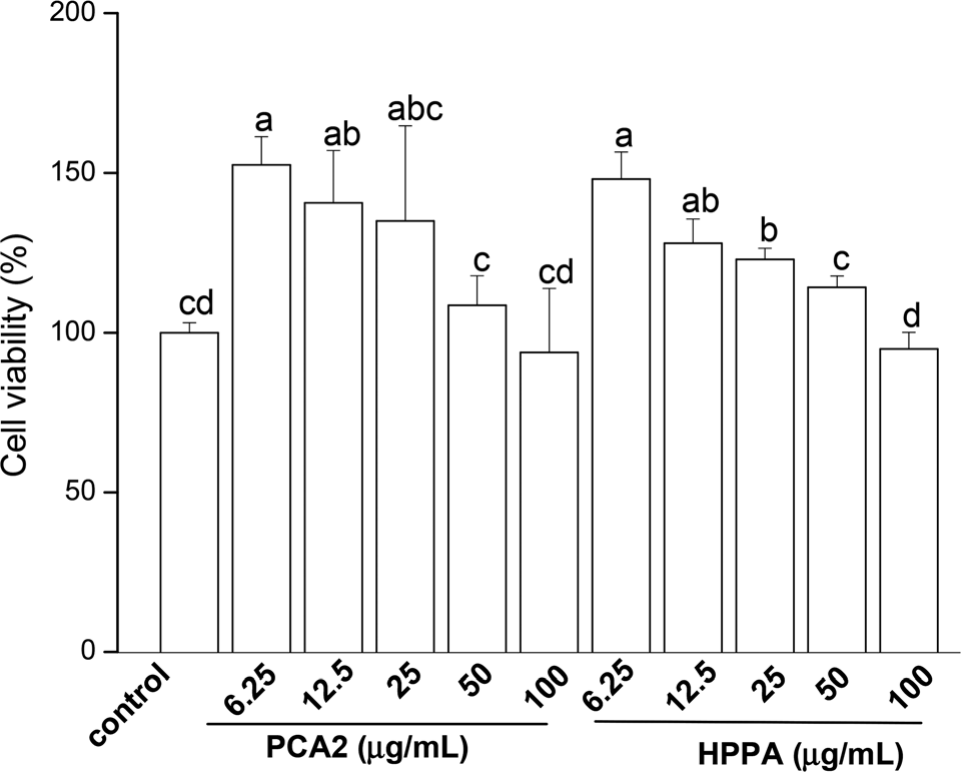
Effects of PCA2 and its major microbial metabolite HPPA on the cell viability of raw264.7 macrophage. The cells were incubated with PCA2 and metabolite HPPA at the concentrations of 6.25, 12.5, 25, 50 and 100 μg mL^−1^ for 24 h. Data are mean ± SD from four independent experiments. Values with different superscript letters above the bar are significantly different (*P* < 0.05).

CCK-8 assay was conducted to assess the effects of PCA2 and the metabolites HPPA on the viability of raw264.7 macrophages. As shown in Fig. 1, compared to control cells, the cell growth was significantly stimulated by PCA2 and the metabolites HPPA within tested concentrations. These results showed that none of the assayed concentrations of PCA2 and HPPA were cytotoxic to macrophages.

### 3.2. PCA2 and the metabolite HPPA inhibited ox-LDL-induced foam cell formation and lipid accumulation

**Fig. 2:**
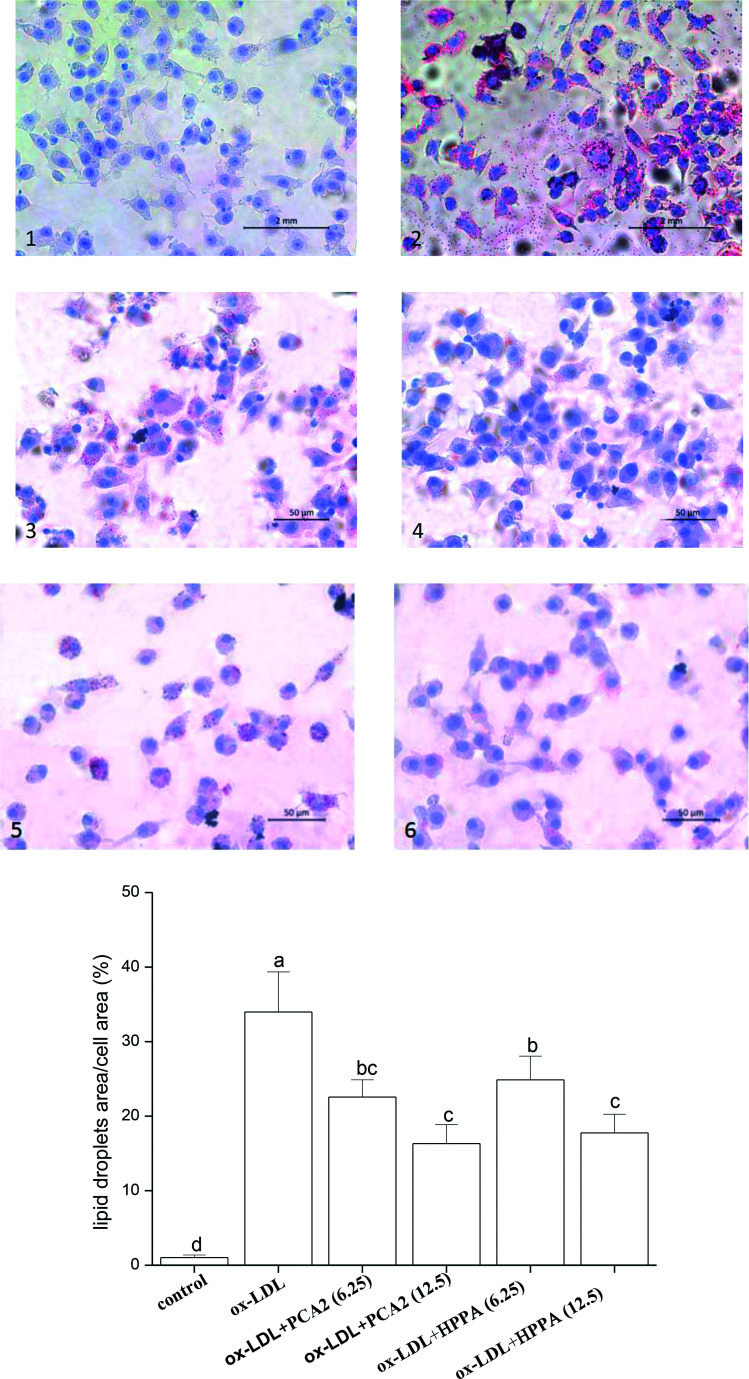
Oil red O staining of RAW264.7 cells incubated with 80 μg mL^−1^ ox-LDL in the presence or absence of PCA2 and HPPA for 48 h (×400). (1) Control, (2) ox-LDL, (3) ox-LDL + 6.25 μg mL^−1^ PCA2, (4) ox-LDL + 12.5 μg mL^−1^ PCA2, (5) ox-LDL + 6.25 μg mL^−1^ HPPA, and (6) ox-LDL + 12.5 μg mL^−1^ HPPA.

As shown in Fig. 2A, the oil-red O staining results were negative for the normal macrophages, while there was heavy reddish staining, and the lipid accumulation was significantly induced after the macrophages were exposed to 80 μg mL^−1^ ox-LDL for 24 h. However, treatment with PCA2 and the microbial metabolites HPPA dose-dependently diminished the reddish lipid droplets. The quantitative levels of the oil-red O staining exhibited that 12.5 μg mL^−1^ PCA2 and HPPA decreased the area of lipid droplets and cell area ratio by 51.96% and 47.72% compared with the model cells, respectively, which indicated that the formation of foam cells was blocked by PCA2 and HPPA.

As shown in Table 2, TC, FC, and CE in the macrophages were significantly increased after exposure to 80 μg mL^−1^ ox-LDL for 24 h, and the ratio of CE to TC reached 54.70%, which means the conversion of macrophages to foam cells. When the macrophages were simultaneously treated with ox-LDL and different concentration of PCA2 and HPPA, the intracellular TC, FC, CE, and CE/TC levels decreased. 12.5 μg mL^−1^ PCA2 significantly decreased the levels of intracellular TC, FC, CE, and CE/TC of model cells by 31.36%, 11.77%, 47.55% and 23.64%, respectively. Likewise, treatment with 12.5 μg mL^−1^ HPPA partially precluded ox-LDL-induced increase in intracellular TC, FC, CE and CE/TC levels by 28.75%, 6.59%, 44.15% and 21.63%, respectively. These data revealed that PCA2 and its microbial metabolite HPPA decreased lipid deposition, and therefore restrained the formation of foam cells induced by ox-LDL.

**Table 2:** Effects of PCA2 and metabolites 4-HPAA on intracellular cholesterol accumulation a(units: mg g^−1^ protein)

Groups	TC	FC	CE	CE/TC (%)
Control	59.52 ± 6.17^d^	41.05 ± 1.62^c^	18.46 ± 7.52^d^	31.02 ± 10.14^d^
ox-LDL	138.97 ± 13.70^a^	62.95 ± 1.14^a^	76.03 ± 13.21^a^	54.70 ± 4.09^a^
ox-LDL + 6.25 μg mL^−1^ PCA2	112.33 ± 2.39^b^	55.51 ± 2.50^b^	56.79 ± 3.25^b^	47.51 ± 2.10^ab^
ox-LDL + 12.5 μg mL^−1^ PCA2	95.39 ± 1.63^c^	55.54 ± 1.80^b^	39.88 ± 0.95^c^	41.77 ± 1.67^c^
ox-LDL + 6.25 μg mL^−1^ HPPA	114.11 ± 1.56^b^	56.56 ± 1.46^b^	55.31 ± 2.68^b^	48.47 ± 1.83^ab^
ox-LDL + 12.5 μg mL^−1^ HPPA	99.02 ± 2.84^c^	58.80 ± 1.58^b^	42.46 ± 4.11^c^	42.87 ± 2.94^bc^

a Values are expressed as mean ± SD. Values in the same column with different superscripts are significantly different at *P* < 0.05 level

### 3.3. PCA2 and the metabolite HPPA protected against ox-LDL-induced oxidative stress

**Fig. 3:**
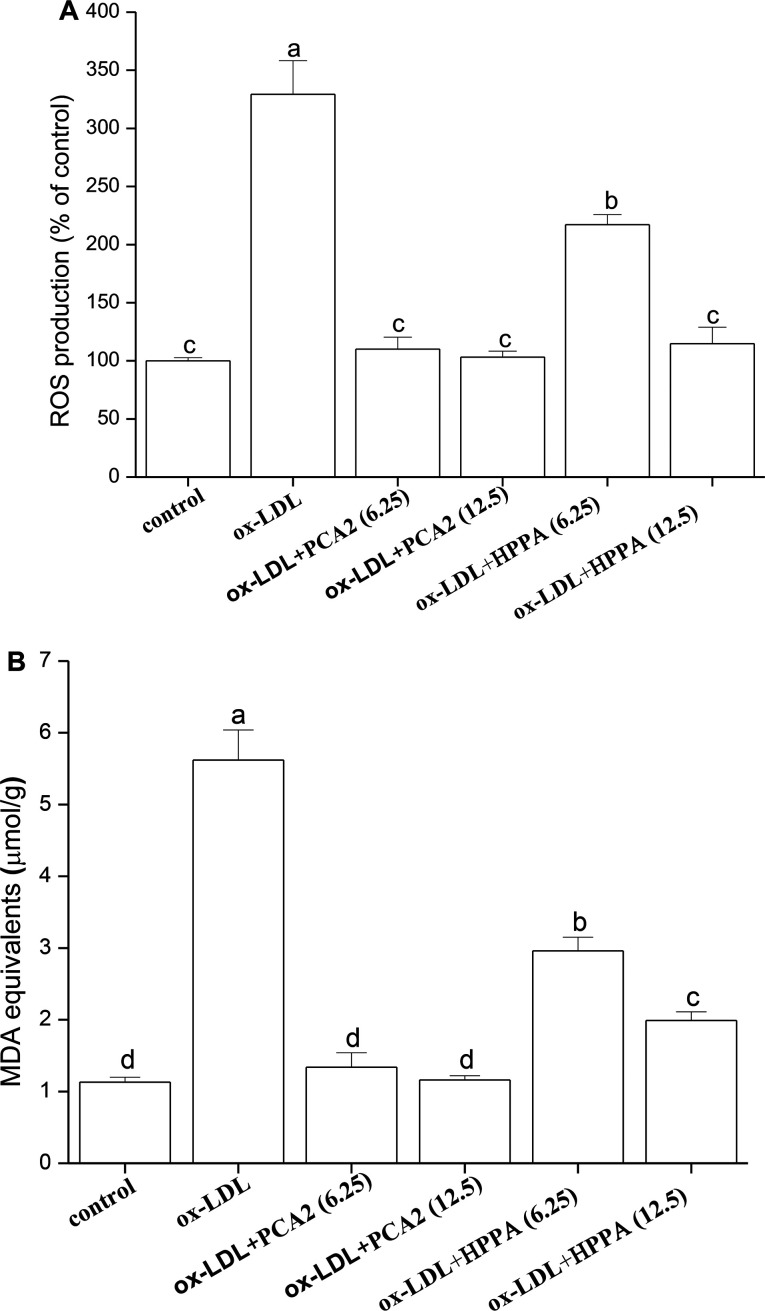
Effect of PCA2 and HPPA on ox-LDL-induced oxidative stress in macrophage cells. Macrophages were treated with 80 μg mL^−1^ ox-LDL in the presence or absence of PCA2 and metabolite HPPA for 24 h. Values with different superscript letters above the bar are significantly different (*P* < 0.05).

The incubation of macrophage cells with 80 μg mL^−1^ ox-LDL for 24 h resulted in significant increases in the levels of ROS and MDA equivalent. These effects were reversed by 6.25 and 12.5 μg mL^−1^ PCA2 treatment (Fig. 3A and B). Microbial metabolites HPPA were less effective preventing the ox-LDL-induced ROS and MDA equivalent level, with only partial recovery of MDA equivalent levels back to those in control cells.

### 3.4. PCA2 and the metabolite HPPA inhibited the secretion of IL-6 and IL-1β

**Fig. 4:**
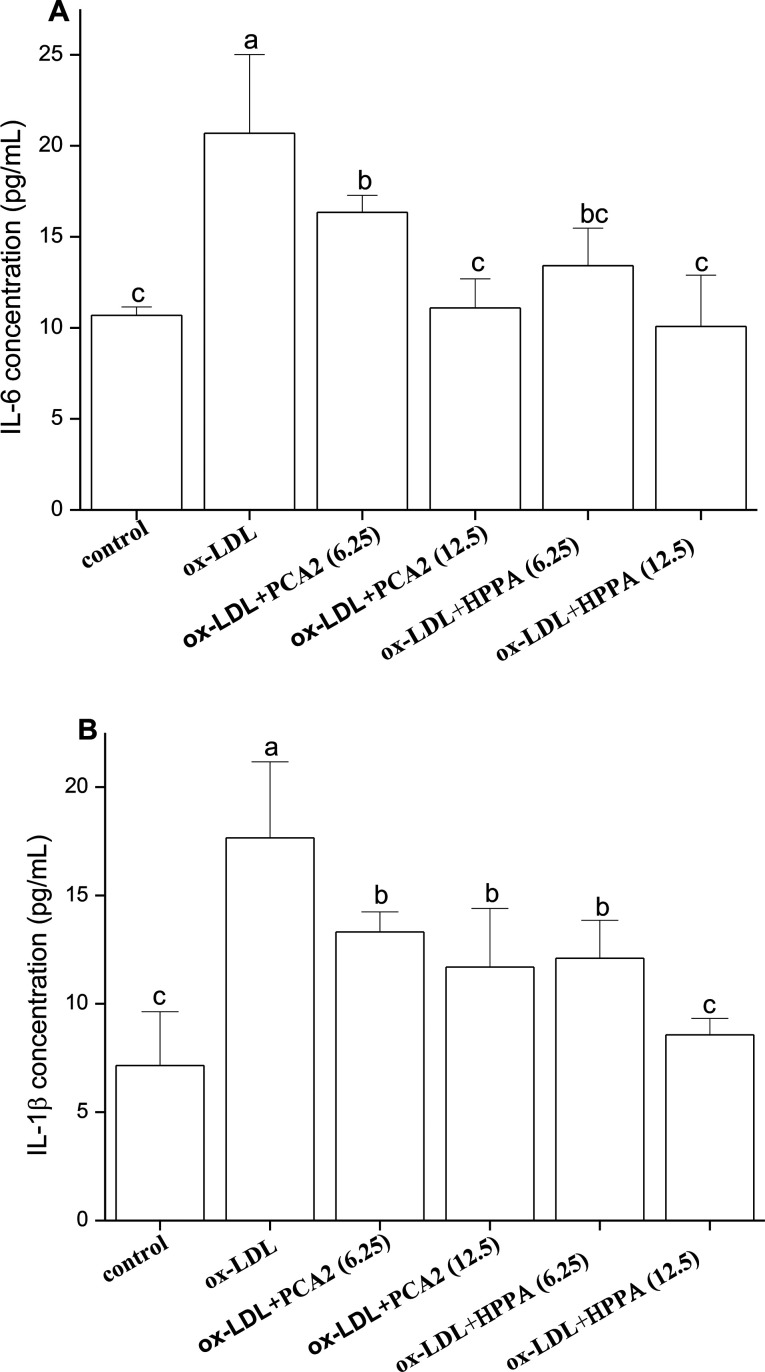
Effects of PCA2 and HPPA on the secretion of inflammatory cytokines. The RAW264.7 cells were treated with 80 μg mL^−1^ ox-LDL in the presence or absence of PCA2 and HPPA for 48 h. (A) IL-6 in culture medium (B) IL-1β in culture medium. Data are mean ± SD from three independent experiments.

As shown in Fig. 4, ox-LDL promoted the production of IL-6 and IL-1β of macrophages. Compared with the model group, PCA2 significantly suppressed the ox-LDL-induced secretion of IL-6 and IL-1β, with a dose-dependent manner (*P* < 0.05). Macrophage IL-6 and IL-1β productions with 12.5 μg mL^−1^ PCA2 treatment were decreased by 46.39% and 33.75% than that of model group, respectively. 12.5 μg mL^−1^ HPPA totally inhibited ox-LDL-induced increase in intracellular IL-1β and IL-6 levels.

### 3.5. Effects of PCA2 and the metabolite HPPA on the mRNA expression of cholesterol efflux/influx-related genes: ABCA1, ABCG1, SR-B1, LXR-α, PPARγ and CD36 in Raw264.7 foam cells

**Fig. 5:**
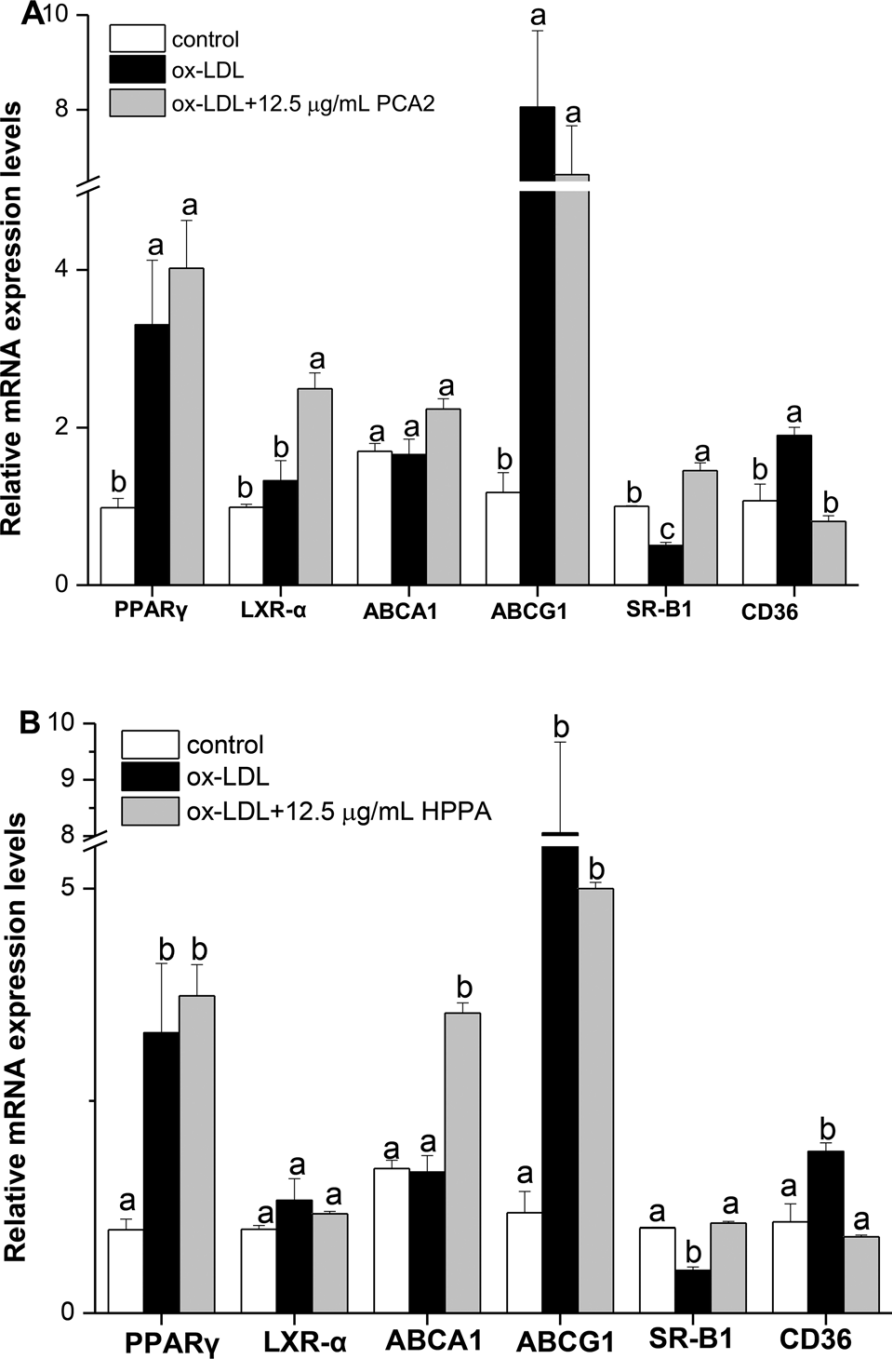
Effect of PCA2 and HPPA on cholesterol efflux/influx-related genes in Raw264.7 foam cells determined by real time PCR. Macrophages were treated with 80 μg mL^−1^ ox-LDL in the presence or absence of PCA2 (A) and HPPA (B) for 10 h. Total RNA was isolated and RT-PCR was performed to examine relative gene expression levels of PPARγ, LXR-α, SR-B1, ABCA1, ABCG1 and CD36. Results represent the mean ± SD of three samples. Different letters above the same column differ significantly (*P* < 0.05).

Cholesterol flux in macrophages is tightly regulated by several genes. ABCA1, ABCG1, SR-B1, LXR-α and PPARγ are key regulators of cholesterol efflux, while CD36 is critical scavenger receptors involved in cholesterol uptake. As shown in Fig. 5, 12.5 μg mL^−1^ PCA2 seperately increased the mRNA levels of LXR-α and SR-B1 by 1.88 and 2.89 times compared with the model group. 12.5 μg mL^−1^ HPPA significantly up-regulated the mRNA levels of ABCA1 and SR-B1 responsible for the efflux of cellular cholesterol. The expression levels of ABCA1 and SR-B1 treatment by 12.5 μg mL^−1^ HPPA were 2.13 and 2.10 times more than the model group, respectively. In addition, the ox-LDL significantly increased the mRNA level of CD36 in RAW264.7 cells, which is recognized as the macrophage mediating the ox-LDL uptake. However, PCA2 and HPPA remarkably down-regulated CD36 mRNA level by 58.99% and 57.42%, respectively.

### 3.6. PCA2 and the metabolite HPPA inhibited the activation of NF-κB pathway induced by ox-LDL

**Fig. 6:**
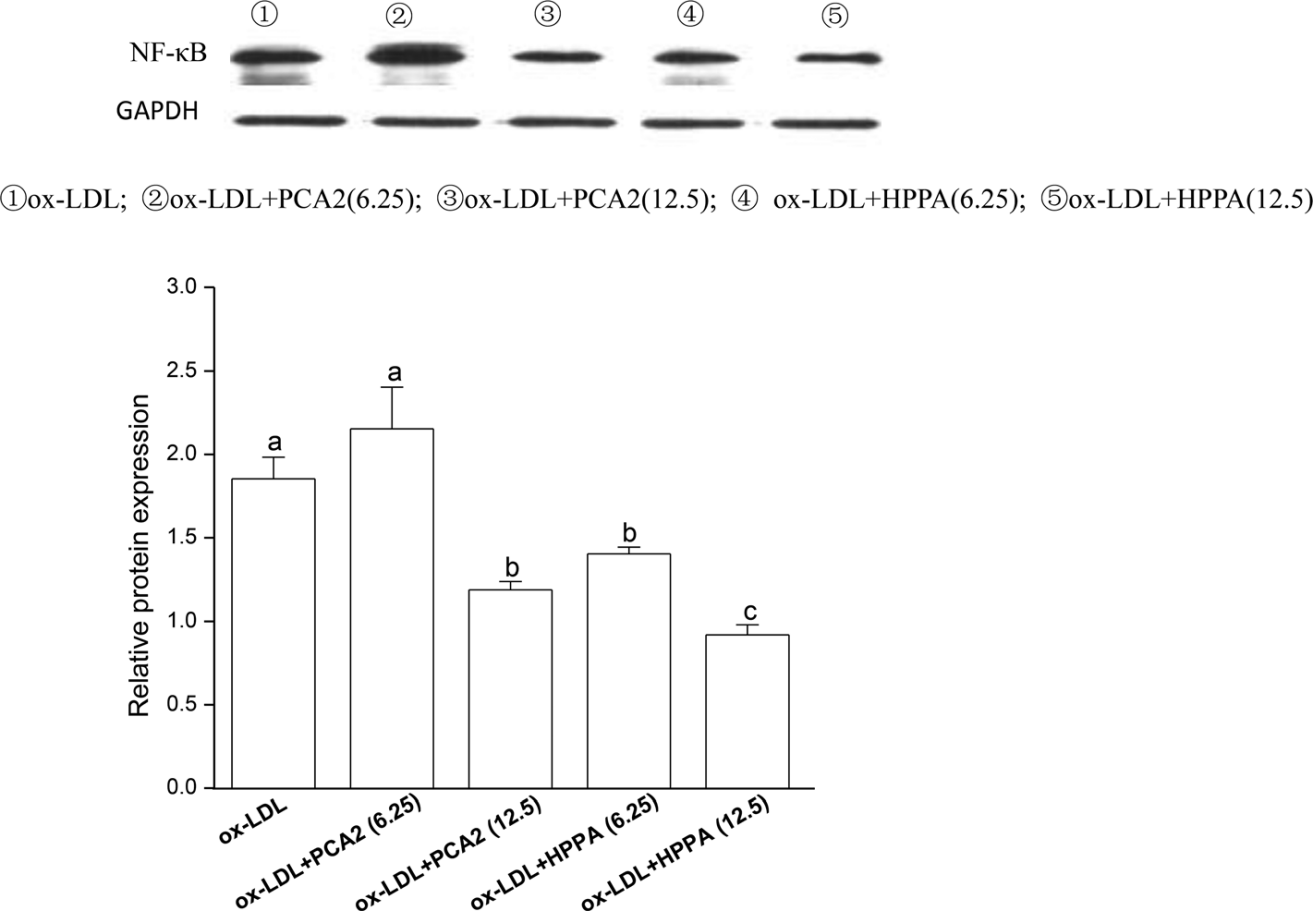
Effect of PCA2 and its metabolite HPPA on protein expression of NF-κB evaluated by western blot in RAW264.7 cells. The RAW264.7 cells were treated with 80 μg mL^−1^ ox-LDL in the presence or absence of PCA2 and HPPA for 24 h. Data are mean ± SD from three independent experiments. Values with different superscript letters above the bar are significantly different (*P* < 0.05).

To further study the possible mechanisms that are responsible for PCA2 and the major metabolite HPPA action, the NF-κB protein expression was analyzed by western blot. As shown in Fig. 6, 12.5 μg mL^−1^ PCA2 or the metabolite HPPA-treated foam cells showed a marked decrease in NF-κB protein expression, showing PCA2 and the metabolite HPPA can inhibit the activation of NF-κB signaling pathway induced by ox-LDL.

## 4. Discussion

Excessive lipids accumulation in macrophages can lead to the formation of foam cells, where the foam cells formation plays a crucial role in promoting AS. It is well accepted that generation of macrophage-derived foam cells is highly associated with the imbalance of cholesterol influx, esterification, and efflux.^32^ Therefore, cholesterol outflow from macrophages, the first step in RCT, has been suggested to play a vital role in the early stage of AS to lower its occurrence.^1^ ABCA1, ABCG1 and SR-B1 have been reported to play the key role in RCT pathway.^33^ Compared to the cells treated with ox-LDL alone, HPPA exhibited good ability to enhance the mRNA levels of ABCA1 and SR-B1, while PCA2 significantly up-regulated SR-B1 and LXR-α mRNA expression levels. Furthermore, CD36 is the principal receptor responsible for the uptake of lipoprotein-derived cholesterol.^34^ We initially observed that ox-LDL increased lipid accumulation associated with the elevated levels of CD36 mRNA level. The increase in CD36 caused by the ox-LDL was significantly decreased by PCA2 and its major microbial metabolite HPPA. Consistent with our results, procyanidins derived from grape seed (mainly composed of B-type procyanidins) attenuate the development of foam cell formation by reducing cholesterol accumulation and modulating the expression of CD36 and ABCA1 expression.^15^ Taken together, the results suggested that PCA2, an A-type procyanidin and its major microbial metabolite regulated the balance between cholesterol uptake and efflux through modulating gene expressions of CD36, ABCA1, SR-B1 and LXR-α, which play important roles in the transformation of macrophages into foam cells. The present work suggested that the inhibition of foam cell formation might be an important mechanism of PCA2 and its major microbial metabolite blocking AS.

It has transpired that the pathogenesis of AS involves a network of vascular wall cells and mediators, in which macrophages play pivotal roles by producing proinflammatory cytokines and *via* transition to lipid-laden foam cells that initiate the formation of atherosclerotic lesion.^35,36^ NF-κB signaling pathways are well accepted to participate in inflammatory cytokine productions and regulate the transcription of downstream targets genes involved in AS.^37^ Ample evidence supports a central regulatory role of NF-κB pathway in atherogenesis.^38^ Our results showed that PCA2 and its major microbial metabolite significantly decreased the ox-LDL-increased levels of IL-6 and IL-1β. Procyanidin B2 inhibits inflammasome activation and IL-1β secretion through the inactivation of the NF-κB signaling pathway in lipopolysaccharide-stimulated macrophages.^39^ Terra *et al.*, reported that grape-seed procyanidins significantly inhibited the overproduction of NO and PGE(2) and NF kappa B (p65) translocation to nucleus in RAW264.7 macrophages stimulated with lipopolysaccharide plus interferon-gamma.^40^ Consistent with these data, our results revealed that PCA2 and its major microbial metabolite significantly suppressed the activation of NF-κB pathways induced by ox-LDL, confirming the fact that inhibition of NF-κB can reduce foam cell formation.

Oxidative stress plays a critical role in the pathogenesis of diverse cardiovascular diseases including AS.^31,41^ Increasing evidence has revealed that ox-LDL can be phagocytosed by macrophages, resulting in macrophage activation and subsequent production of inflammatory cytokines and ROS.^42,43^ Therefore, suppression of ox-LDL-induced oxidative stress may be an efficient strategy to prevent and minimize the development of AS. Many antiatherosclerotic therapeutics exert their atheroprotective effects by inhibiting macrophage oxidative stress.^44,45^ Previous studies have shown a protective effect of PCA2 against oxidative stress, protecting against bisphenol A-induced apoptosis in islet cells and decreasing the MDA equivalents level in pancreas tissue.^46^ Similarly, our data also revealed that PCA2 and its major metabolite HPPA protected against ox-LDL-induced oxidative stress. HPPA was less effective than PCA2 and only partly moderating the oxidative stress induced by ox-LDL, not being able to recover the cellular MDA equivalents level completely.

Current evidences indicate that a large proportion of ingested procyanidins would reach the colon, where they undergo metabolism by the intestinal microbiota generating a wide variety of metabolites. As mentioned above, it has been reported that the bioavailability of PCA2 is low,^47^ generating metabolites including 5-phenylvaleric acid, 4-hydroxyphenylacetic acid, 3-(3-hydroxyphenyl)propionic acid and HPPA which is the major metabolites.^30,48^ Therefore, it is crucial to assess whether its metabolites retain the biological activity of the parent compounds. In the present work the microbial metabolite HPPA showed a protective effect against foam cells formation similar to that of PCA2, whilst other metabolites was less effective (data not shown). The results implied that the protective effect of PCA2 against foam cells formation is strengthened by the action of its major microbial metabolite. To the best of our knowledge, this is the first report on the atheroprotective activities of PCA2 and its major microbial metabolites in a foam cell model.

## 5. Conclusion

**Fig. 7:**
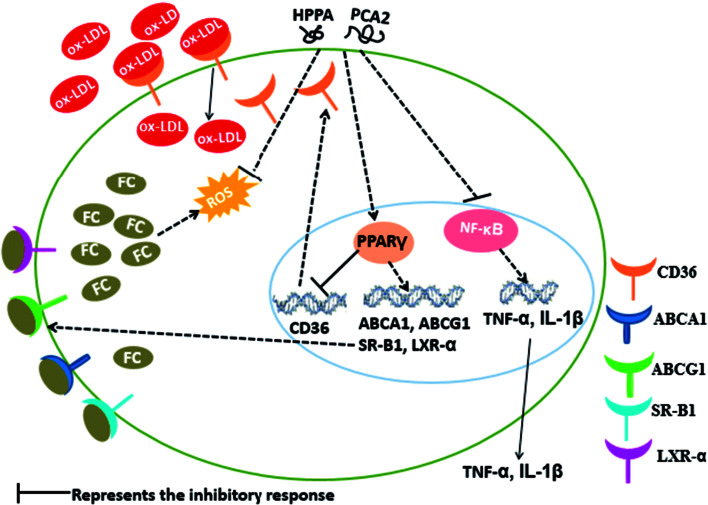
Possible molecular mechanism of PCA2 and its metabolite HPPA-mediated suppression of macrophage foam cell formation.

In summary, our study showed that PCA2, an A-type procyanidin and its major microbial metabolites, HPPA, effectively hinder the formation of foam cells. The cells treated with PCA2 or its major microbial metabolites were protected from the deleterious effect of ox-LDL as shown by the lower lipids accumulation, decreased ROS and MDA equivalents production, and diminished levels of inflammatory biomarkers (Fig. 7). The microbial metabolite HPPA showed a similar suppression effect on the macrophage foam cell formation with that of PCA2. The protective effect of PCA2 against foam cells formation might partly attribute to its microbial metabolites.

## Conflicts of interest

There are no conflicts to declare.

## Supplementary Material
